# *ERG11* Gene Mutations and *MDR1* Upregulation Confer Pan-Azole Resistance in Candida tropicalis Causing Disseminated Candidiasis in an Acute Lymphoblastic Leukemia Patient on Posaconazole Prophylaxis

**DOI:** 10.1128/AAC.02496-16

**Published:** 2017-06-27

**Authors:** Liangshun You, Wenbin Qian, Qing Yang, Liping Mao, Li Zhu, Xianbo Huang, Jie Jin, Haitao Meng

**Affiliations:** a Department of Hematology, the First Affiliated Hospital, College of Medicine, Zhejiang University, Hangzhou, People's Republic of China; b State Key Laboratory for Diagnosis and Treatment of Infectious Diseases, Collaborative Innovation Center for Diagnosis and Treatment of Infectious Diseases, First Affiliated Hospital, College of Medicine, Zhejiang University, Hangzhou, People's Republic of China

**Keywords:** *ERG11* mutation, *MDR1*, azole resistance, Candida tropicalis, candidiasis, posaconazole, acute leukemia

## Abstract

In this study, we present a rare case of fatal breakthrough Candida tropicalis infection in a patient with acute lymphoblastic leukemia (ALL) while on posaconazole prophylaxis. Then, we explore the mechanisms underlying azole resistance by focusing on enhanced efflux pumps and changes in the azole target enzyme Erg11p, which was encoded by the ERG11 gene. Our study demonstrates that Y132C substitution of Erg11p combined with MDR1 overexpression may be the pan-azole resistance mechanisms in Candida tropicalis.

## TEXT

The incidence of and mortality rate caused by systemic fungal infection has increased steadily over the past 30 years, mainly associated with antifungal resistance (1). As recent advances in oncology and supportive care have extended the survival of patients with hematologic malignancy, invasive fungal diseases (IFDs) are now the largest infectious challenge in these patients (2, 3). Posaconazole is a new oral triazole with broad-spectrum antifungal activity that has been used as both antifungal prophylaxis and salvage therapy against IFDs in high-risk patients (4). However, the breakthrough of IFDs in patients who received posaconazole prophylaxis remains a challenging problem (5–9). The main mechanisms of azole resistance in Candida species are associated with enhanced efflux pumps and changes in the azole target enzyme Erg11p (10, 11). Here, we report a rare case of fatal breakthrough Candida tropicalis infection while on posaconazole prophylaxis and explore the molecular basis for the azole resistance mechanisms.

**FIG 1:**
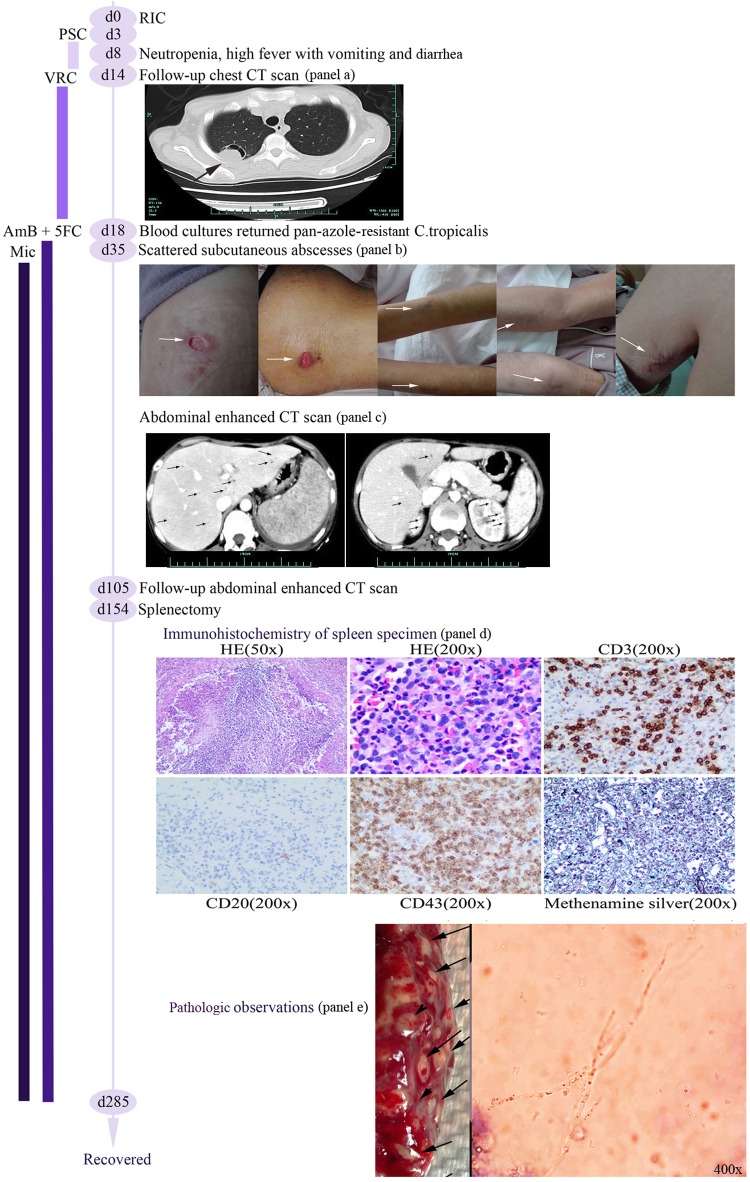
Schematic presentation of the patient's clinical course. RIC, remission-induction chemotherapy; PSC, posaconazole; VRC, voriconazole; AmB, amphotericin B; 5FC, 5-flucytosine; Mic, micafungin.

A female patient in her early 30s was admitted to the First Affiliated Hospital of Zhejiang University with a first diagnosis of B-cell acute lymphoblastic leukemia (ALL) in August 2015. She was started on remission-induction chemotherapy on the same day (day 0) and received oral posaconazole prophylaxis (200 mg three times per day) on day 3. A consecutive episode of febrile neutropenia (day 8) was treated with empirical broad-spectrum antibiotics, but high fever with severe vomiting and diarrhea persisted. The antifungal agent was changed from posaconazole to voriconazole (200 mg every 12 h). A follow-up chest computed tomography (CT) scan (day 14) showed a large lung nodule with an air crescent sign (Fig. 1a), which was strongly suggestive of fungal pulmonary infection. Meanwhile, pan-azole resistant Candida tropicalis was isolated from blood cultures at three different times (days 18, 28, and 30). On day 18, antifungal agents were switched to intravenous amphotericin B (AmB; MIC, 1 μg/ml) at a dose of 1 mg/kg/day and combined with 5-flurocytosine (5FC; MIC, 4 μg/ml) at a dose of 2.5 g every 12 h.

Scattered subcutaneous abscesses spread throughout the body on day 35 (Fig. 1b), and an abdominal enhanced CT scan showed diffused small abscesses in the liver, the upper poles of both kidneys, and the whole spleen (Fig. 1c). Drainage fluid from subcutaneous abscesses and necrotic tissue cultures both returned pan-azole resistant Candida tropicalis with the same sensitivity tests as the isolates from blood cultures. The antifungal agent micafungin (Mic) at 150 mg per day was added at the appearance of subcutaneous abscesses. The patient gradually recovered from the subcutaneous abscesses, but high fever persisted, and a follow-up abdominal enhanced CT scan on day 105 showed aggravated multiple organ abscesses, especially in the spleen. Splenectomy was performed after multidisciplinary team consultation. Dense abscesses were found in the whole spleen, and budding cells with pseudohyphae consistent with candida infection could be seen under the microscope after an India ink stain (Fig. 1e). Immunohistochemistry showed leukocyte common antigen (LCA) and was positive for CD3/CD43 (T cells) and negative for CD20 (B cells), while a Gömöri methenamine silver stain was positive (Fig. 1d), all characteristic of Candida infection. On day 285, the patient had recovered and was in complete remission from ALL.

The different Candida tropicalis isolates from the patient were identified by the CHROMAgar Candida API 20C system and confirmed by large-subunit (26S) rDNA gene sequences. Susceptibility tests were performed in accordance with CLSI M27-A3 and M27-S4 guidelines, and the MICs were interpreted according to the documented CLSI breakpoints (12). The results showed that all isolates from the patient were identical and were characterized by a high resistance to fluconazole (MIC, 256 μg/ml), itraconazole (MIC, 4 μg/ml), voriconazole (MIC, 16 μg/ml), and posaconazole (MIC, 2 μg/ml).

**FIG 2:**
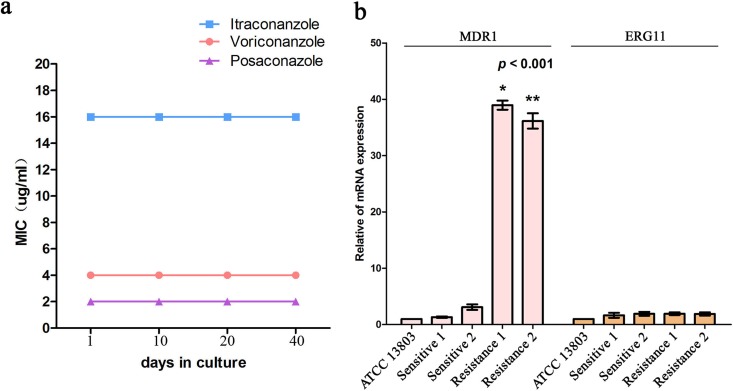
(a) Assessment of the stability of azole resistance *in vitro*: all pan-azole resistant C. tropicalis strains isolated from patients were serially cultured in drug-free medium, and then itraconazole (ITC), voriconazole (VRC), and PSC MICs were assayed after 20 subcultures (2 days per subculture), which showed the same results. Results are representative of all strain experiments (*n* = 3 independent experiments). (b) RT-qPCR analysis of *MDR1* and *ERG11* mRNA expression: *MDR1* relative gene expression levels in pan-azole-resistant strains (one from blood culture, and the other from necrotic tissue culture) showed significant overexpression in comparison with the susceptible strains *, *P* < 0.001; **, *P* < 0.001 (*n* = 3 independent experiments). Error bars indicate the standard deviation (SD).

In order to assess the stability of azole resistance *in vitro*, all Candida tropicalis strains were serially cultured in drug-free medium, and then itraconazole, voriconazole, and posaconazole MICs were assayed after 20 subcultures. The results indicated that the azole resistance could not be reversed (Fig. 2a).

Since the overexpression of multidrug efflux transporters and targeted enzyme Erg11p have been known to be involved in azole resistance in Candida albicans (10), these molecular mechanisms were explored in our study. The analysis of relative expression of resistance gene mRNA in the pan-azole resistant strains with real-time PCR showed significant overexpression of the *MDR1* gene in comparison with the two susceptible strains (*P* < 0.001) (Fig. 2b). However, this was not the case for the other two associated resistance genes, *CDR1* (data not shown) and *ERG11* (Fig. 2b). *MDR1*-homologous genes have been found in different species of Candida, and the correlation between *MDR1* upregulation and the fluconazole resistance phenotype has been previously demonstrated (13). From our own and previous studies, we infer that *MDR1* overexpression may be one of the azole resistance mechanisms in these pan-azole resistant Candida tropicalis strains.

Several *ERG11* gene mutations have been previously described to be associated with azole resistance in Candida tropicalis (14); therefore, the *ERG11* genes of the pan-azole resistant strains were sequenced and aligned with the online published wild-type sequences (GenBank accession no. XM_002550939.1) using MUSCLE software. Interestingly, double homozygous mutations of A395T and C461T, resulting in the substitution of Y132C and S154C in the amino acid sequence of Erg11p, were detected in all pan-azole-resistant strains.

**FIG 3:**
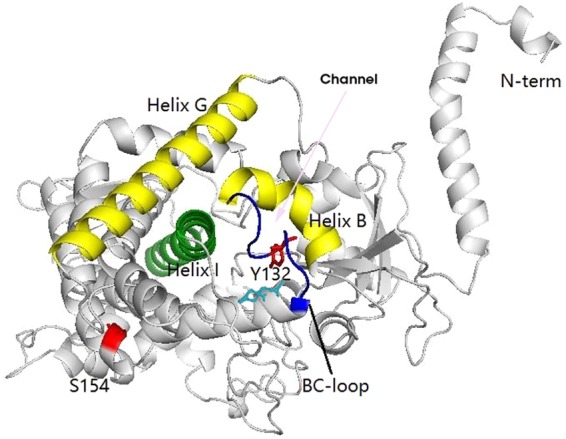
Three-dimensional model of the Erg11p enzyme of Candida tropicalis: we first searched for the most similar structure using PDB-blast (http://www.pdb.org) and found PDB ID 5JLC to have the highest score. Therefore, we conducted homology modeling of the three-dimensional structure of Erg11p by SWISS-MODEL using 5JLC as the template. Then, the protein structures were visualized by Pymol software. The pink arrow represents the substrate access channel; navy blue, the BC loop; cyan, heme iron; red, mutation sites Y132 and S154.

To investigate the relationship between azole resistance and the mutations in the target enzyme, we mapped the two mutation sites in the three-dimensional model of Erg11p, of which the Y132C substitution was in the B-C loop (Fig. 3), which localized in the mouth of the substrate access channel. The mutations in the B-C loop would decrease the affinity of Erg11p for azoles, which has been proven by previous studies to result in azole resistance (14, 15). In addition, the S154C substitution was located at the surface of Erg11p; therefore, it is unlikely to be associated with azole resistance (Fig. 3). Hence, the Y132C substitution in Erg11p may confer pan-azole resistance in these Candida tropicalis strains.

In summary, the breakthrough of IFDs in patients receiving posaconazole prophylaxis remains a challenging problem (16, 17). Our study details a rare case of fatal breakthrough Candida tropicalis causing disseminated candidiasis during posaconazole prophylaxis in a patient with ALL, and we found that the mutation Y132C in Erg11p associated with *MDR1* upregulation may confer pan-azole resistance in Candida tropicalis.

## References

[1] Silva NC, Nery JM, Dias AL 2014 Aspartic proteinases of *Candida* spp.: role in pathogenicity and antifungal resistance. Mycoses 57:1–11. doi:10.1111/myc.12095. 23735296

[2] Chamilos G, Luna M, Lewis RE, Bodey GP, Chemaly R, Tarrand JJ, Safdar A, Raad II, Kontoyiannis DP 2006 Invasive fungal infections in patients with hematologic malignancies in a tertiary care cancer center: an autopsy study over a 15-year period (1989–2003). Haematologica 91:986–989. 16757415

[3] Marr KA, Carter RA, Crippa F, Wald A, Corey L 2002 Epidemiology and outcome of mould infections in hematopoietic stem cell transplant recipients. Clin Infect Dis 34:909–917. doi:10.1086/339202. 11880955

[4] Nagappan V, Deresinski S 2007 Reviews of anti-infective agent posaconazole a broad-spectrum triazole antifungal agent. Clin Infec 45:1610–1617. doi:10.1086/523576. 18190324

[5] Ullmann AJ, Lipton JH, Vesole DH, Chandrasekar P, Langston A, Tarantolo SR, Greinix H, Morais de Azevedo W, Reddy V, Boparai N, Pedicone L, Patino H, Durrant S 2007 Posaconazole or fluconazole for prophylaxis in severe graft-versus-host disease. N Engl J Med 356:335–347. doi:10.1056/NEJMoa061098. 17251530

[6] Cornely OA, Maertens J, Winston DJ, Perfect J, Ullmann AJ, Walsh TJ, Helfgott D, Holowiecki J, Stockelberg D, Goh YT, Petrini M, Hardalo C, Suresh R, Angulo-Gonzalez D 2007 Posaconazole vs. fluconazole or itraconazole prophylaxis in patients with neutropenia. N Engl J Med 356:348–359. doi:10.1056/NEJMoa061094. 17251531

[7] Auberger J, Lass-Flörl C, Aigner M, Clausen J, Gastl G, Nachbaur D 2012 Invasive fungal breakthrough infections, fungal colonization and emergence of resistant strains in high-risk patients receiving antifungal prophylaxis with posaconazole: real-life data from a single-centre institutional retrospective observational study. J Antimicrob Chemother 67:2268–2273. doi:10.1093/jac/dks189. 22653819

[8] Hahn J, Stifel F, Reichle A, Holler E, Andreesen R 2011 Clinical experience with posaconazole prophylaxis—a retrospective analysis in a haematological unit. Mycoses 54:12–16. doi:10.1111/j.1439-0507.2010.01980.x. 21126267

[9] Lerolle N, Raffoux E, Socie G, Touratier S, Sauvageon H, Porcher R, Bretagne S, Bergeron A, Azoulay E, Molina JM, Lafaurie M 2014 Breakthrough invasive fungal disease in patients receiving posaconazole primary prophylaxis: a 4-year study. Clin Microbiol Infect 20:O952–O959. doi:10.1111/1469-0691.12688. 24861577

[10] Noël T 2012 The cellular and molecular defense mechanisms of the *Candida* yeasts against azole antifungal drugs. J Mycol Med 22:173–178. doi:10.1016/j.mycmed.2012.04.004. 23518020

[11] Heilmann CJ, Schneider S, Barker KS, Rogers PD, Morschhäuser J 2010 An A643T mutation in the transcription factor Upc2p causes constitutive *ERG11* upregulation and increased fluconazole resistance in *Candida albicans*. Antimicrob Agents Chemother 54:353–359. doi:10.1128/AAC.01102-09. 19884367 PMC2798556

[12] Pfaller MA, Diekema DJ 2012 Progress in antifungal susceptibility testing of *Candida* spp. by use of Clinical and Laboratory Standards Institute broth microdilution methods, 2010 to 2012. J Clin Microbiol 50:2846–2856. doi:10.1128/JCM.00937-12. 22740712 PMC3421803

[13] Barchiesi F, Calabrese D, Sanglard D, Falconi Di Francesco L, Caselli F, Giannini D, Giacometti A, Gavaudan S, Scalise G 2000 Experimental induction of fluconazole resistance in *Candida tropicalis* ATCC 750. Antimicrob Agents Chemother 44:1578–1584. doi:10.1128/AAC.44.6.1578-1584.2000. 10817712 PMC89916

[14] Sanglard D, Ischer F, Koymans L, Bille J 1998 Amino acid substitutions in the cytochrome P-450 lanosterol 14alphademethylase (CYP51A1) from azole-resistant *Candida albicans* clinical isolates contribute to resistance to azole antifungal agents. Antimicrob Agents Chemother 42:241–243. doi:10.1093/jac/42.2.241. 9527767 PMC105395

[15] Kelly SL, Lamb DC, Kelly DE 1999 Y132H substitution in *Candida albicans* sterol 14alpha-demethylase confers fluconazole resistance by preventing binding to haem. FEMS Microbiol Lett 180:171–175. doi:10.1111/j.1574-6968.1999.tb08792.x. 10556708

[16] Pagano L, Caira M, Candoni A, Aversa F, Castagnola C, Caramatti C, Cattaneo C, Delia M, De Paolis MR, Di Blasi R, Di Caprio L, Fanci R, Garzia M, Martino B, Melillo L, Mitra ME, Nadali G, Nosari A, Picardi M, Potenza L, Salutari P, Trecarichi EM, Tumbarello M, Verga L, Vianelli N, Busca A, SEIFEM Group. 2012 Evaluation of the practice of antifungal prophylaxis use in patients with newly diagnosed acute myeloid leukemia: results from the SEIFEM 2010-B registry. Clin Infect Dis 55:1515–1521. doi:10.1093/cid/cis773. 22955439

[17] Bellanger AP, Albert ND, Lewis RE, Walsh TJ, Kontoyiannis DP 2015 Effect of preexposure to triazoles on susceptibility and virulence of *Rhizopus oryzae*. Antimicrob Agents Chemother 59:7830–7832. doi:10.1128/AAC.01583-15. 26392499 PMC4649224

